# Randomized phase 2 study of gemcitabine and cisplatin with or without vitamin supplementation in patients with advanced esophagogastric cancer

**DOI:** 10.1007/s00280-018-3588-6

**Published:** 2018-04-25

**Authors:** A. A. van Zweeden, C. J. van Groeningen, R. J. Honeywell, E. Giovannetti, R. Ruijter, C. H. Smorenburg, G. Giaccone, H. M. W. Verheul, G. J. Peters, Hans J. van der Vliet

**Affiliations:** 1 Department of Medical Oncology, VU University Medical Center, Amsterdam, The Netherlands; 2 Department of Internal Medicine, Amstelland Hospital, Amstelveen, The Netherlands; 3 Department of Internal Medicine, Noordwest Ziekenhuisgroep Alkmaar, Alkmaar, The Netherlands; 4 Department of Medical Oncology, Georgetown University Medical Center, Washington, DC USA; 5 Department of Medical Oncology, VU University Medical Center, Room 3A38, De Boelelaan 1117, 1081 HV Amsterdam, The Netherlands

**Keywords:** Esophagogastric cancer, Cisplatin, Gemcitabine, Vitamin B12, Folic acid

## Abstract

**Purpose:**

Preclinical research and prior clinical observations demonstrated reduced toxicity and suggested enhanced efficacy of cisplatin due to folic acid and vitamin B12 suppletion. In this randomized phase 2 trial, we evaluated the addition of folic acid and vitamin B12 to first-line palliative cisplatin and gemcitabine in patients with advanced esophagogastric cancer (AEGC).

**Methods:**

Patients with AEGC were randomized to gemcitabine 1250 mg/m^2^ (i.v. days 1, 8) and cisplatin 80 mg/m^2^ (i.v. day 1) q 3 weeks with or without folic acid (450 µg/day p.o.) and vitamin B12 (1000 µg i.m. q 9 weeks). The primary endpoint was response rate (RR). Secondary endpoints included overall survival (OS), time to progression (TTP), toxicity, and exploratory biomarker analyses. Cisplatin sensitivity and intracellular platinum levels were determined in adenocarcinoma cell lines cultured under high and low folate conditions in vitro.

**Results:**

Adenocarcinoma cells cultured in medium with high folate levels were more sensitive to cisplatin and this was associated with increased intracellular platinum levels. In the randomized phase 2 clinical trial, which ran from October 2004 to September 2013, treatment was initiated in 78 of 82 randomized pts, 39 in each study arm. The RR was similar; 42.1% for supplemented patients vs. 32.4% for unsupplemented patients; *p* = 0.4. Median OS and TTP were 10.0 and 5.9 months for supplemented vs. 7.7 and 5.4 months for unsupplemented patients (OS, *p* = 0.9; TTP, *p* = 0.9). Plasma homocysteine was lower in the supplemented group [*n* = 20, 6.9 ± 1.6 (mean ± standard error of mean, SEM) µM; vs. 12.5 ± 4.0 µM; *p* < 0.001]. There was no significant difference in the *C*_max_ of gemcitabine and cisplatin in the two treatment groups.

**Conclusion:**

Folic acid and vitamin B12 supplementation do not improve the RR, PFS, or OS of cisplatin and gemcitabine in patients with AEGC.

## Introduction

Esophagogastric cancer is one of the most common malignancies of the gastrointestinal tract worldwide. These cancers encompass malignant epithelial neoplasms located in all regions of the esophagus and stomach irrespective of the histological type. In the majority of cases, the malignancies are adenocarcinomas (AC) or squamous cell carcinomas (SCC). The incidence of SCC has largely remained constant over time, while the incidence of AC has increased [1]. Treatment for metastatic disease is palliative and frequently consists of combination chemotherapy and/or radiotherapy. The goals of palliative systemic chemotherapy are survival benefit and palliation of symptoms [2, 3]. Cisplatin has been considered a key substance in combination regimens for metastatic gastro-esophageal cancer [4]. Results from a phase 2 study at our institute showed a response rate (RR) of 41% using the combination of cisplatin and gemcitabine, with manageable toxicity [5]. Treatment with pemetrexed plus cisplatin and vitamin supplementation resulted in superior survival time, time to progression, and response rates compared with treatment with cisplatin alone in patients with malignant mesothelioma in the EMPHACIS trial [6]. The majority of patients in this study received folic acid and vitamin B12. Vitamin suppletion significantly reduced toxicity of the chemotherapy and did not decrease efficacy parameters. Vitamin suppletion was found to be predictive of increased overall survival in a multivariate regression analysis of prognostic factors derived from this trial [7]. Preclinical evidence demonstrated that differences in the folate environment resulted in a different sensitivity of human cancer cell lines to cisplatin [8, 9]. Tumor cells that are relatively cisplatin resistant require lower intracellular folate concentrations for growth [10]. In line, low tumor cell expression levels of the folate receptor (FR), which is a major influx transporter for folates in normal tissues and certain tumors [11], are associated with cisplatin resistance [12, 13]. In this paper, we first investigated the cisplatin sensitivity of adenocarcinoma cell lines grown under high or low folate conditions. Adenocarcinoma cells grown under high folate conditions were more sensitive to cisplatin and this was associated with higher intracellular platinum accumulation, providing a rationale for supplementation of patients with folates. Based on these in vitro data and the clinical suggestion of increased efficacy in mesothelioma patients we hypothesized that folate supplementation to patients would increase the sensitivity to cisplatin-based treatment. We designed a randomized phase 2 trial to determine whether supplementation of folic acid and vitamin B12 could increase the efficacy of gemcitabine and cisplatin in advanced esophagogastric cancer.

## Patients and methods

### Effect of folic acid supplementation on intratumoral accumulation of cisplatin and tumor cell sensitivity to cisplatin

To determine whether and how folic acid supplementation would affect sensitivity to cisplatin we tested the cisplatin sensitivity of two pairs of adenocarcinoma cell lines WiDr and CaCo-2 and their sublines (WiDr/LF, CaCo-2/LF/LV and CaCo-2/LF/FA) adapted to grow under low folate conditions [13, 14]. Due to the unavailability of modified esophageal adenocarcinoma cell lines, adenocarcinoma cell lines of colorectal origin were used for this purpose. Standard mycoplasma testing was performed. Wild type WiDr and CaCo-2 are cultured in standard DMEM medium containing 8 µM folic acid, WiDr/LF and CaCo-2/LF/LV have been selected to grow in folate-free RPMI medium supplemented with 2.5 and 1 nM leucovorin, respectively, while CaCo-2/LF/FA is adapted to grow in RPMI medium supplemented with 1 nM folic acid. Sensitivity of these cells to cisplatin was determined by a 72 h exposure to cisplatin alone or in combination with gemcitabine using the sulforodamide B (SRB) assay [15]. We also determined whether folate supplementation would affect the accumulation of cisplatin into these cells. Intracellular platinum concentrations were determined as described earlier [16].

### Clinical study design and study population

The clinical study was a multicenter randomized open label phase 2 study comparing therapy with gemcitabine and cisplatin with or without vitamin B12 and folic acid supplementation. From October 2004 to August 2013, 82 patients were included in the study. The study recruited patients in the VU University medical center (VUmc) in Amsterdam, The Netherlands and the Noordwest Ziekenhuisgroep in Alkmaar, The Netherlands. Main inclusion criteria included histologically or cytologically confirmed metastatic or locally advanced unresectable advanced esophagogastric carcinoma (AEGC), squamous cell or adenocarcinoma, not amenable to curative treatment, measurable disease according to RECIST [17], age of at least 18 years, ECOG performance score of 0–2, life expectancy of at least 12 weeks, adequate bone marrow function, adequate renal function, and adequate hepatic function. Prior surgery, chemotherapy and/or radiotherapy in the neo-adjuvant or adjuvant setting was allowed as long as the chemotherapy was completed at least 6 months prior to entry of the study. Written informed consent was obtained from all patients prior to inclusion into the study. Patients with known symptomatic metastasis in the central nervous system (CNS) or suffering from any serious concomitant systemic disorders incompatible with study treatment were not eligible. Other exclusion criteria were treatment with any investigational agent in the month prior to inclusion or prior diagnosis of other malignant disease (excluding adequately treated in situ carcinoma of the cervix and non-melanoma skin cancer, low grade prostate carcinoma or any other non-relapsed malignancy that was treated more than 5 years before diagnosis). Randomization was performed by the data management center of the Integraal Kanker Center Amsterdam (IKA) using a computerized randomization system. The institutional Medical Ethical board of the VUmc and Noordwest Ziekenhuisgroep approved the trial, which was in accordance with the Declaration of Helsinki and Good Clinical Practice.

### Study treatment

Patients were randomized to receive treatment with gemcitabine 1250 mg/m^2^ intravenously (i.v.) on days 1 and 8 in combination with cisplatin 80 mg/m^2^ i.v. on day 1 in a 3 weekly cycle with or without vitamin supplementation, further described as supplemented vs. unsupplemented patients, respectively.

Vitamin supplementation consisted of folic acid 450 µg/24 h per os (p.o.), starting at least 1 week prior to chemotherapy and finishing at least 3 weeks after the last treatment dose, and vitamin B12 1000 µg (1 vial intramuscularly, i.m.) every 9 weeks, starting 1 week before chemotherapy and finishing at least 3 weeks after the last treatment dose.

Patients were treated with up to six cycles of chemotherapy. Study treatment was discontinued in case of progressive disease, unacceptable toxicity or upon patient request.

### Endpoints

The primary endpoint of this study was to determine whether supplementation of folic acid and vitamin B12 could increase the response rate (RR) of patients with advanced esophagogastric cancer treated with the combination of gemcitabine and cisplatin. Secondary endpoints were assessment of time to progression (TTP), defined as the time from randomization to progression and overall survival (OS), defined as the time from randomization to death. Further secondary objectives included the assessment of plasma homocysteine concentrations as an indication for plasma folic acid homeostasis. Moreover, we investigated the effect of folate supplementation on plasma pharmacokinetics of cisplatin (total and free unbound), gemcitabine, the gemcitabine degradation product 2′,2′-difluoro-2′-deoxyuridine (dFdU) and, in white blood cells (WBC), its active metabolite gemcitabine–triphosphate (dFdCTP). We also determined polymorphisms in the genes encoding methylenetetrahydrofolate reductase (MTHFR), which may affect folate homeostasis [11], and cytidine deaminase (CDA), that catalyzes the deamination of gemcitabine to dFdU [18].

### Toxicity evaluation, dose adjustments and response assessment

Treatment toxicity was rated according to CTC version 2.0 (CTCAE v2.0 Cancer Therapy Evaluation Program, Common Terminology Criteria for Adverse Events, Version 2.0, DCTD, NCI, NIH, DHHS) (http://ctep.cancer.gov). All serious adverse events (SAE) were collected from registration until 30 days after the last protocol treatment administration. Criteria for chemotherapy administration on day 1 of each cycle was delayed 1 week in case of neutropenia (absolute neutrophil count (ANC) of < 1.5 × 10^9^/L) or thrombopenia (platelets < 100 × 10^9^/L), renal toxicity (creatinine > 120 µmol/L, and/or creatinine clearance < 60 mL/min) or any non-haematological toxicity above CTC grade 1 or baseline. Gemcitabine was reduced on day 8 with 25 or 50% in case of grade 2 or 3 neutropenia or grade 1 or 2 thrombopenia, respectively. Platelets < 50 × 10^9^/L or neutrophils < 0.5 × 10^9^/L were reason to omit gemcitabine on day 8. The dose of gemcitabine was reduced 50% in case of grade 3 non-haematological toxicity (except emesis) and discontinued in case of grade 4 AE’s. The doses of gemcitabine and cisplatin were reduced with 25% after a 2 week treatment delay due to toxicity, neutropenic fever, grade 4 neutropenia and/or thrombopenia lasting over 1 week or thrombopenia associated with bleeding. Cisplatin was reduced or discontinued in case of grade ≥ 2 peripheral neuropathy or grade ≥ 2 renal toxicity or other grade ≥ 3 non-haematological toxicity (except emesis). No dose escalations were allowed. Unacceptable toxicity was defined as failure to recover from side effects after a treatment delay of a maximum of 3 weeks, requirement of a third dose reduction, the repeated occurrence of grade 3 or 4 non-haematological toxicity or drug-induced pneumonitis ≥ grade 2 or according to investigator’s judgement. Tumor assessments by CT scan of chest and abdomen were performed every 6 weeks until disease progression according to RECIST [17]. A baseline scan was done within 4 weeks before initiation of study therapy. Disease status, date of progression, date of death and subsequent lines of therapy were collected during regular follow-up visits. TTP was defined as the time from randomization to progression. OS was defined as the time from randomization to death.

### Assessment of potential predictive parameters

Plasma pharmacokinetics of gemcitabine, its metabolite dFdU and cisplatin were measured during treatment in the first 20 patients to assess whether vitamin supplementation would affect either gemcitabine or cisplatin pharmacokinetics. We also determined the concentration of dFdCTP in WBC and the homocysteine concentration in these 20 patients. The other patients were monitored for homocysteine before randomization and at the beginning of each 3rd chemotherapy cycle (1 week after vitamin B12 administration). To assess these parameters, blood was collected in heparinized tubes containing tetrahydrouridine to prevent conversion of gemcitabine to dFdU. After centrifugation the plasma was taken off and stored at − 20 °C until analysis. The intermediate layer between plasma and red blood cells containing the WBC was layered on Ficoll–Hypaque, centrifuged and the buffy coat with the WBC was washed, counted and the pellet was frozen in liquid nitrogen until analysis for dFdCTP. Gemcitabine, dFdU and dFdCTP were measured with validated HPLC assays [16]. Homocysteine was measured as described earlier [19]. To determine the amount of total and free (non-protein) bound platinum species, one part was immediately frozen at − 20 °C until analysis (total platinum), and the other part was mixed with ethanol, incubated overnight at − 20 °C, and centrifuged. The supernatant contained free platinum [20]. Free plasma platinum and total plasma platinum (free and protein-bound platinum) were determined using flameless atomic absorption spectroscopy [16, 20].

The 79A > C (rs2072671) CDA and 667C > T MTHFR polymorphism were analyzed in, respectively, 37 and 20 patients in this study to asses a possible association with response, survival and toxicity.

### Statistical analysis

Based on a hypothesized 1.5-fold improvement in the RR from an anticipated 33% in the non-supplemented arm to 50% in the vitamin supplemented arm, a sample size of 82 patients (41 per study arm) was required. If the true RR difference between the study regimens would be ≥ 15%, there would be an approximate 90% probability of selecting the true superior arm. The unpaired *t* test was used to compare RR and the stratified log-rank test was used to compare survival rates between treatment groups. An intention to treat analysis was used for TTP and OS. OS and TTP were calculated using Kaplan–Meier estimates. The correlation between homocysteine levels in both treatment groups was determined with a 2-tailed *t* test. *p* values < 0.05 were considered statistically significant.

## Results

### High folate status increases sensitivity to cisplatin

**Fig. 1:**
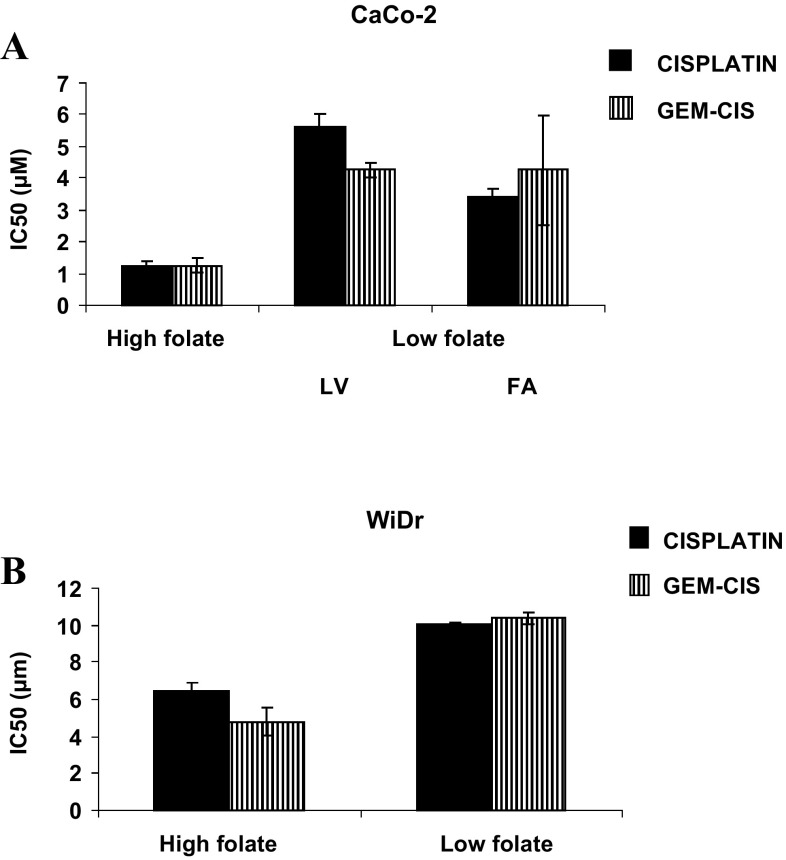
High folate conditions can increase sensitivity of adenocarcinoma cells to cisplatin. CaCo-2 and WiDr adenocarcinoma cell lines were treated with cisplatin and gemcitabine under high folate or low folate conditions. **a** CaCo-2 and sublines CaCo-2/LF/LV and CaCo-2/LF/FA; **b** WiDr. CaCo-2-LF sublines CaCo-2-LF/LV (IC_50_ 5.6 ± 0.5 µM; *p* < 0.01), CaCo-2-LF/FA (IC_50_ 3.4 ± 0.3 µM; *p* < 0.02), and WiDr/LF (IC_50_ 10.1 ± 0.1 µM; *p* < 0.02) under low folate (LF) conditions were two to five fold less sensitive to cisplatin compared to culture under high folate conditions. *LF* low folate, *FA* folic acid reduced into the nM range, *LV* leucovorin reduced into the nM range

Under normal (high folate) cell culture conditions CaCo-2 cells were more sensitive to cisplatin than WiDr cells (IC_50_ 1.2 ± 0.2 µM for CaCo-2 vs. 6.4 ± 0.5 µM for WiDr, means ± SEM, *p* < 0.001). However, when cultured under low folate (LF) conditions these cell lines were two to five fold less sensitive to cisplatin (IC_50_ for CaCo-2-LF sublines CaCo-2-LF/LV, 5.6 ± 0.5 µM; *p* < 0.01 and CaCo-2-LF/FA 3.4 ± 0.3 µM; *p* < 0.02, and for WiDr/LF 10.1 ± 0.1 µM; *p* < 0.02), as shown in Fig. 1. The addition of gemcitabine to cisplatin resulted in a slight increase in cisplatin sensitivity, but the difference in IC_50_ between high and low folate containing medium remained the same.

To investigate the mechanism behind the observed difference in cisplatin sensitivity of tumor cells cultured in medium containing different folate concentrations, tumor cells were exposed to 20 µM cisplatin for 24 h. WiDr cells accumulated more platinum than WiDr-LF cells (50 ± 0.5 vs. 28 ± 3 pmol/10^6^ cells, respectively; *p* < 0.0001), while CaCo-2 cells (96 ± 16 pmol/10^6^ cells) accumulated more platinum than CaCo-2-LF/LV (58 ± 8 pmol/10^6^ cells; *p* < 0.05) or CaCo-2-LF/FA (44 ± 6 pmol/10^6^ cells; *p* < 0.02). Co-incubation with gemcitabine resulted in a slight increase in cisplatin accumulation, especially under LF conditions (not shown). Overall, these data demonstrate that high folate conditions can increase sensitivity of adenocarcinoma cells to cisplatin, and that this is associated with higher intratumoral platinum accumulation, providing a rationale for supplementation of patients with folates.

### Patient characteristics

A total of 82 patients were randomly assigned to each treatment arm (41 patients in each arm). Baseline characteristics were generally well matched between the two treatment arms (Table 1). The mean age of patients was 61 years (range 35–83). The majority of patients were male, and most (72%) suffered from advanced esophageal cancer. In 85–90% of patients the ECOG performance score was 0–1. Less than 10% of the supplemented patients and none of the unsupplemented patients had undergone prior treatment for esophagogastric carcinoma (two patients received palliative radiotherapy of the primary tumor, one patient received neo-adjuvant chemotherapy and one patient received prior chemoradiation). Treatment was initiated in 78 patients. Four patients did not receive chemotherapy after randomization. One patient, allocated to the vitamin group, deceased unexpectedly before the first chemotherapy, while another patient in the same treatment group was not eligible due to neutropenia. Two patients allocated to the treatment arm without vitamin suppletion were not eligible due to increasing renal impairment. These four patients could not be monitored for response but were included in the intention to treat analysis for TTP and OS.

**Table 1:** Baseline patient and disease characteristics

	Supplemented patients *N* = 41	Unsupplemented patients *N* = 41
Characteristic		
Mean age (range)	61 year (50–78)	61 (35–82)
Gender (female/male)	8/33	8/33
Primary tumor (stomach/esophagus)	11/30	12/29
Tumor type (SCC/AC)	8/33	8/33
Performance status		
PS 0	14 (34%)	12 (29%)
PS 1	23 (56%)	23 (56%)
PS 2	2 (5%)	4 (10%)
PS unknown	2 (5%)	2 (5%)
Prior therapy	4 (10%)	0

### Safety and tolerability

The overall incidence of grade 3–5 AEs was comparable between the two treatment groups and probably caused by the chemotherapy (Table 2). Grade 3 leukopenia was the most common severe toxicity in supplemented patients (22%), while fatigue was the most common severe toxicity (24%) in unsupplemented patients. Three supplemented patients suffered from grade 4 thrombopenia. Grade 4 thrombopenia was reported in one unsupplemented patient. Two supplemented patients were diagnosed with an ischemic cerebrovascular accident (CVA) after two treatment cycles (grade 4 neurologic toxicity) and one patient was diagnosed with a hemorrhagic CVA three days after day 1 of the first chemotherapy cycle (grade 4 hemorrhage) and received no further study treatment. Two unsupplemented patients deceased shortly after the first chemotherapy cycle, in 1 case probably due to cardiac arrhythmias likely caused by cardiac metastases and in the other case due to the occurrence of cardiac failure. These events were considered to be most likely related to cisplatin chemotherapy and underlying predisposing conditions of the patients. These three patients could not be monitored for response but were included in the intention to treat analysis for PFS and OS.

**Table 2:** Treatment related grade 3–5 AEs per study arm

Adverse event	Supplemented patients (*n* = 41), *n* (%)			Non-supplemented patients (*n* = 41), *n* (%)		
	Grade			Grade		
	3	4	5	3	4	5
Febrile neutropenia	2 (5)			1 (2)		
Leukopenia	9 (22)			4 (10)		
Trombopenia	4 (10)	3 (7)		4 (10)	1 (2)	
Anemia	6(15)			2 (5)		
Fatigue	4 (10)			10 (24)		
Cardiac	1 (2)			1 (2)		2 (5)
Neurologic	1 (2)	2 (5)		5 (12)		
Ototoxicity				1 (2)		
Pulmonary				1 (2)		
Nausea	4 (10)			3 (7)		
Vomiting	2 (5)			2 (5)		
Anorexia	2 (5)			5 (12)		
Liver	1 (2)					
Diarrhea				1 (2)		
Pain				1 (2)		
Skin	1 (2)					
Renal/bladder	5 (12)					
Hemorrhage	1 (2)	1 (2)		2 (5)		
Infection	1 (2)			1 (2)		

Percentages are rounded to whole numbers. For each grade 3/4/5 adverse event the maximum toxicity was noted per patient

Twenty patients (in both treatment groups) were treated with darbepoetin alfa for chemotherapy induced anemia [21] with a hemoglobin response in 15 of these 20 patients. Darbepoetin did not increase toxicity.

### Response rate, overall survival and time to progression

#### Response rate

Thirty-eight supplemented patients and 37 unsupplemented patients were evaluable for response. The RR was 42.1% (*n* = 16) for supplemented patients, all partial responses (PR). The RR for unsupplemented patients was 32.4% (*n* = 12), and consisted of PR in 29.7% (*n* = 11) and a complete response (CR) in 2.7% (*n* = 1) of patients. The RR was not significantly different between the two treatment groups, *p* = 0.4.

### Overall survival and time to progression

**Fig. 2:**
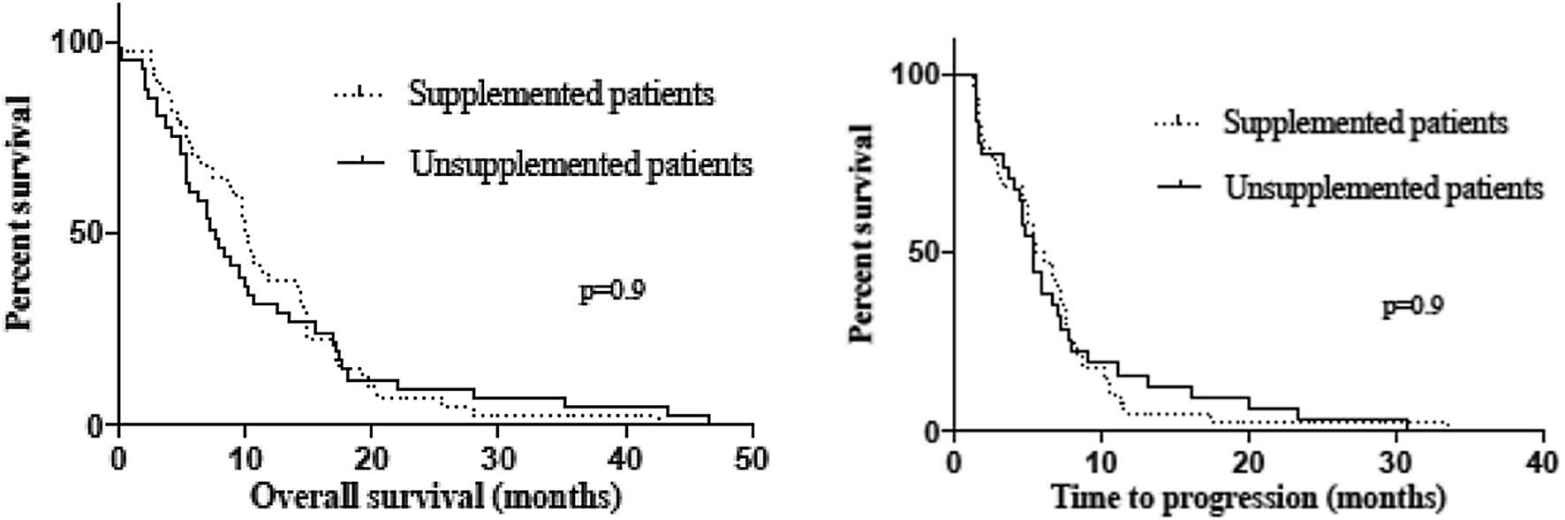
Kaplan–Meier curve for OS and TTP. The dotted line represents the supplemented arm, while the black line represents the unsupplemented arm. OS and TTP were not significantly different between the supplemented vs. the unsupplemented patients (median OS 10.0 months; range 0.3–42.6 vs. 7.7 months; range 0.03–46.7; *p* = 0.9; median TTP 5.9 months; range 1.4–33.5 vs. 5.4 months; range 1.4–30.9; *p* = 0.9)

The median OS in this study was 10.0 months (range 0.3–42.6) for the supplemented arm vs. 7.7 months (range 0.03–46.7) for the unsupplemented arm. This difference was not statistically significant (*p* = 0.9). One patient was lost to follow-up and was censored for the OS analysis. According to the prespecified intention to treat analysis all other randomized patients were included in the survival analysis, including one patient who deceased between randomization and the start of study treatment and one patient who was treated with epirubicin and oxaliplatin instead of cisplatin–gemcitabine due to renal impairment. This patient was not included in the TTP analysis. Vitamin supplementation did not lead to a significantly different median TTP, 5.9 months (range 1.4–33.5) for supplemented patients vs. 5.4 months (range 1.4–30.9) for unsupplemented patients (*p* = 0.9). The Kaplan–Meier curves for OS and TTP are shown in Fig. 2.

### Pharmacokinetic monitoring and assessment of potential prognostic parameters

**Fig. 3:**
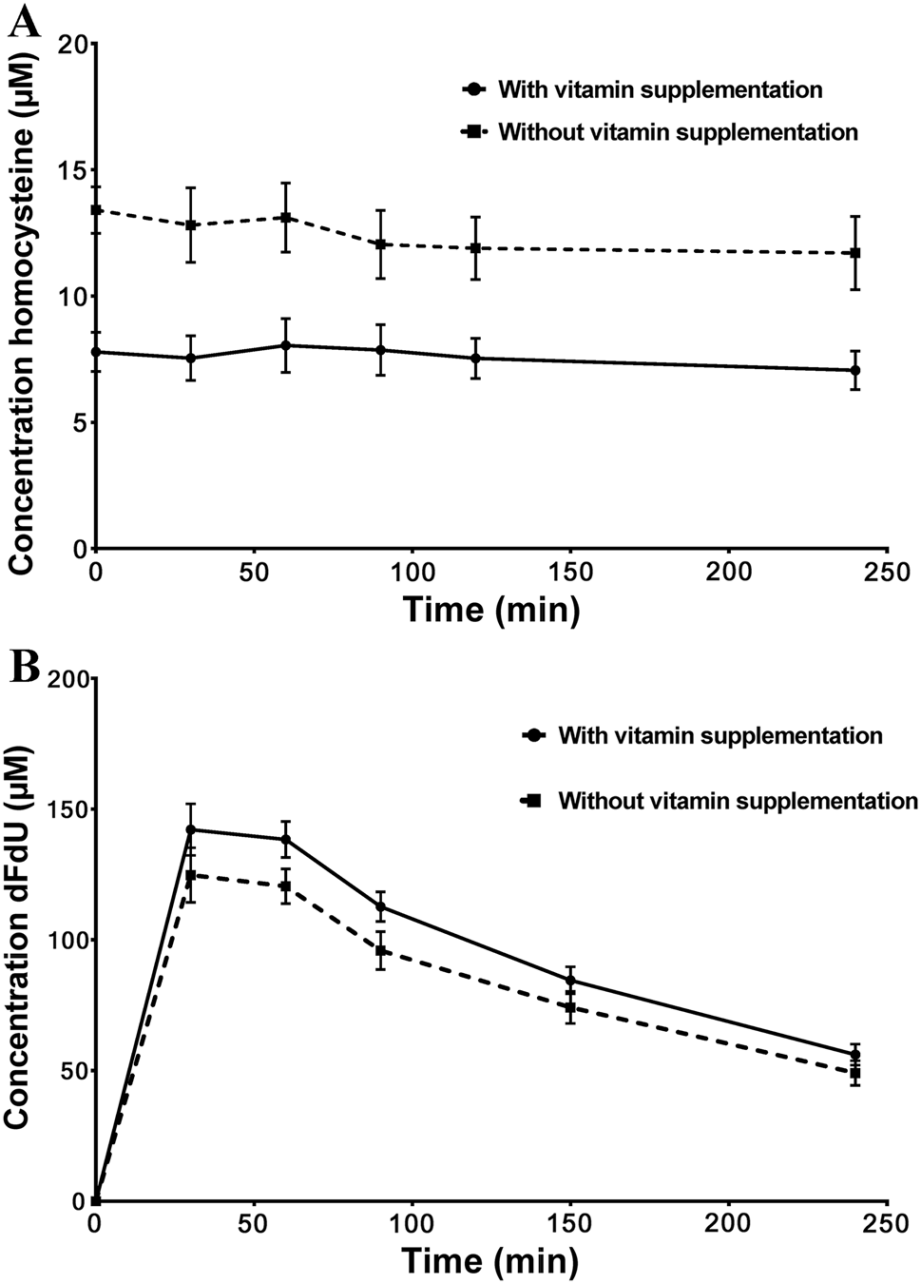
Plasma concentrations of homocysteine and dFdU in vitamin supplemented and unsupplemented the patients from the pharmacokinetics cohort. **a** The black line represents the supplemented arm, while the dotted line represents the unsupplemented arm. Values are means ± SEM from ten patients in each cohort. Homocysteine levels were lower in supplemented patients vs. unsupplemented patients (mean 6.9 ± 1.6 µM; range 6.2–7.2 vs. 12.5 ± 4.0 µM; range 11.7–13.4; *p* < 0.001); **b** The black line represents the supplemented arm, while the dotted line represents the unsupplemented arm. Supplementation resulted in increased levels of the gemcitabine metabolite dFdU (*p* < 0.05). Values are means ± SEM from 10 to 7 patients, respectively

Following vitamin supplementation, homocysteine levels were lower in supplemented patients vs. unsupplemented patients (mean 6.9 ± 1.6 µM; range 6.2–7.2 vs. 12.5 ± 4.0 µM; range 11.7–13.4; *p* < 0.001), as shown in Fig. 3. This difference was expected, since homocysteine levels are inversely related to folate and vitamin B12 consumption. Compared to pre-randomization levels, vitamin supplementation decreased homocysteine levels in supplemented patients, while homocysteine increased when patients were randomized to the unsupplemented arm. This illustrates compliance to the allocated treatment arm. The maximum concentration (*C*_max_) of gemcitabine was identical for patients in both treatment groups (*n* = 20; *C*_max_ 53.4 ± 18.6 (mean ± SEM) µM for supplemented vs. 53.2 ± 15.0 µM for unsupplemented pts). However, vitamin supplementation did change gemcitabine pharmacokinetics. For example, supplementation resulted in increased levels of the gemcitabine metabolite dFdU (Fig. 3; *p* < 0.05), and in addition also resulted in an increase in the formation of the active metabolite of gemcitabine dFdCTP (peak levels at 30 min 255 ± 190 vs. 133 ± 93 pmol/10^6^ cells and at 90 min 298 ± 245 vs. 226 ± 101 pmol/10^6^ cells; *p* > 0.1), though these results were not statistically significant. Vitamin supplementation led to a small, but not statistically significant, increase in total cisplatin levels (total platinum 15.7 ± 2.7 µM in the vitamin group vs. 14.8 ± 1.4 µM for patients without vitamin supplementation), and a significant increase in free (non-protein bound) platinum levels: 4.7 ± 1.7 µM in the vitamin group vs. 3.6 ± 0.7 µM without vitamin supplementation (*p* < 0.05). Genetic polymorphisms in the folate metabolizing enzyme MTHFR 677C > T were measured in 20 patients, while polymorphisms in the gemcitabine metabolizing enzyme CDA 79A > C were measured in 37 patients. For the CDA gene, neither the OS (*p* = 0.56; Log-rank; Mantel–Cox test), TTP (*p* = 0.61; Log-rank; Mantel–Cox test) nor the RR (*p* = 0.46, ANOVA test) differed significantly between the patients with the AA, CC or AC variant, although analyzed patient numbers may be too small to formally rule out smaller differences (Table 3). Similarly, the incidence of grade 3 toxicity of any cause was not statistically significantly different between patients with either of the three polymorphisms (*p* = 0.9).The OS (*p* = 0.90 Log-rank; Mantel–Cox test), TTP (*p* = 0.89 Log-rank; Mantel–Cox test) and RR (*p* = 0.42 ANOVA test) were not significantly different for patients with a TT, CC or CT MTHFR 677. Again numbers may be considered too small for a reliable comparison. Grade 3 toxicity of any cause was equally distributed between the different polymorphisms.

**Table 3:** CDA and MTHFR gene polymorphisms in relation to outcome and toxicity

	AA *n* = 22	CC *n* = 7	AC *n* = 8	TT *n* = 2	CC *n* = 6	CT *n* = 12
Clinical parameter						
OS (months)	7.8 (SD 9.1)	17.3 (SD 13.7)	10.1 (SD 3.1)	11.0 (SD 12.4)	5.0 (SD 19.3)	9.8 (SD 11.0)
TTP (months)	5.5 (SD 12.9)	9.0 (SD 8.6)	6.8 (SD 2.0)	10.5 (−)	1.9 (SD 12.8)	6.4 (SD 9.0)
PR/CR	*n* = 8	*n* = 3	*n* = 5	*n* = 1	*n* = 1	*n* = 6
RR (%)	36	43	63	50	17	50
Grade 3 toxicity	*n* = 12 (55%)	*n* = 4 (57%)	*n* = 5 (63%)	*n* = 1 (50%)	*n* = 3 (50%)	*n* = 6 (50%)
Grade 4 toxicity	–	–	*N* = 1 (13%)	–	–	–

Polymorphisms in the gene for CDA were measured in 37 patients. The AA variant was found in 22 patients, the CC variant in 7 patients and AC variant in 8 patients. Polymorphisms in the gene for MTHFR were measured in 20 patients. The TT variant was found in 2 patients, the CC variant in 6 patients and CT variant in 12 patients

*OS* overall survival (median months), *TTP* time to progression (median months), *RR* response rate (%), *PR* partial response, *CR* complete response, *SD* standard deviation

## Discussion

In this study we demonstrate that the combination of gemcitabine and cisplatin can be considered an effective palliative chemotherapeutic regime in patients with AEGC. The median combined OS of 9.2 months and TTP of 5.4 months in our study is comparable with the currently commonly used first-line palliative chemotherapy regimens for AEGC. The current standard first-line palliative chemotherapy for AEGC consists of triplet chemotherapy regimens such as EOX (epirubicin, oxaliplatin and capecitabine, OS/PFS 11.2/7.0 months), ECX (epirubicin, cisplatin, capecitabine, OS/PFS 9.9/6.7 months) or EOF (epirubicin, oxaliplatin and fluorouracil, OS/PFS 9.3/6.5 months) [22] or doublet therapies (fluorouracil, leucovorin in combination with oxaliplatin or cisplatin, OS/PFS resp. 10.7/5.8 vs. 8.8/3.9 months) [23], or capecitabine and oxaliplatin, OS 8 months [24]. A recent meta-analysis showed a limited survival benefit of triplet chemotherapy with an increased risk of toxicity when compared to doublet chemotherapy [25]. Cisplatin and gemcitabine have a different side effect profile compared with oxaliplatin-based chemotherapy regimens. Cisplatin is associated with a higher incidence of grade 3–4 neutropenia, alopecia, thromboembolism, and renal dysfunction, while peripheral neuropathy and diarrhea is a more frequent side effect of oxaliplatin [26, 27]. The intravenous administration route of cisplatin and gemcitabine can be a relevant consideration for patients with AEGC and problems with the passage of food (and oral medication such as, e.g., capecitabine) as a result of obstruction caused by the primary tumor. Therefore, the here employed cisplatin and gemcitabine treatment combination can be considered a reasonable or perhaps even preferred palliative treatment option for AEGC patients with, e.g., signs of dysphagia or preexistent neuropathy.

This multicenter randomized phase 2 trial was designed to investigate whether supplementation of folic acid and vitamin B12 resulted in an improved clinical outcome in AEGC patients treated with the combination of cisplatin and gemcitabine chemotherapy. The addition of folic acid and vitamin B12 to this chemotherapy backbone did not significantly increase the RR which was 42.1% (*n* = 16) in the vitamin group vs. 32.4% (*n* = 12) in the chemotherapy alone group. The difference in RR between the study arms did not meet the prespecified target RR of 50% nor a 15% difference in RR between the two treatment groups. The median OS was not significantly different with or without vitamin suppletion (10.0 vs. 7.7 months). The median TTP was similar in both treatment groups (5.9 months for supplemented patients vs. 5.4 months for unsupplemented patients). Baseline characteristics of the patients in both study arms were well balanced. Vitamin supplementation did not result in an apparent decrease in the incidence of grade 3–5 adverse events.

As homocysteine levels are inversely related to folate and vitamin B12 consumption, the measured lower concentration of homocysteine in patients receiving concomitant vitamin supplementation is indicative of the biological activity of the employed vitamin supplementation and is in support of adequate patient compliance [28]. Though the use of second line chemotherapy or experimental therapy was not specifically documented in this trial, its use could potentially affect differences in OS between the two study groups. The impact of second line chemotherapy in AEGC was very limited if at all present and is unlikely to substantially confound our data.

Our study results contrast with the previously reported beneficial effects observed when folic acid and vitamin B12 were added to the combination of cisplatin and pemetrexed and cisplatin monotherapy [6]. Apart from the fact that a different patient group was studied, both studies also differed in their design as our study was randomized for the addition of vitamins, while the study of Vogelzang et al. was not randomized for vitamin suppletion. The discrepancy could also be related to specific effects of folate and vitamin B12 on the efficacy of pemetrexed that do not occur with the cisplatin and gemcitabine combination used in this study [29]. Indeed, in the phase 3 study of Vogelzang et al. the benefit of adding vitamin suppletion was predominantly observed in the group of patients treated with pemetrexed and cisplatin. Moreover, as in our study cisplatin was combined with gemcitabine a potential positive effect of folate suppletion on cisplatin sensitivity (e.g., an increased exposure to free platinum) might have been masked by effects on gemcitabine metabolism as, e.g., degradation of gemcitabine to dFdU was increased in supplemented patients. In earlier studies an association between increased gemcitabine deamination and a lower response rate and survival were reported [18]. The formation of the active metabolite of gemcitabine dFdCTP in white blood cells, included as a surrogate biomarker for tissue accumulation, was initially increased. The levels of dFdCTP and the effect of cisplatin are in line with other studies of gemcitabine–cisplatin combination therapy [30]. However, this difference did not persist and may, therefore, preclude a clinically relevant increase of dFdCTP levels in tissues, which is necessary for an optimal effect of gemcitabine [31]. One can also not exclude that the potentiating effects of vitamin supplementation, as found in patients with mesothelioma, differ between tumor types. The 79A > C polymorphism in the CDA gene and the 667C > T polymorphism in the MTHFR gene were measured in a subgroup of patients. We found no correlation with RR, OS, TTP or severe toxicity although numbers were small and the study was not powered for this analysis.

The results of this trial are important for daily practice, since vitamin supplement use is very common among patients with cancer [32, 33]. Reasons for vitamin suppletion include an expected reduced toxicity of chemotherapy, an expected enhanced efficacy of cancer treatment in combination with vitamin use and an expected improvement in general well-being. Complementary medicine is very often not evidence based [34], but in this randomized trial no efficacy benefit was found of vitamin suppletion. In recent years, clinical trials for advanced esophagogastric cancer have focused more on triple-drug regimens. These consist of chemotherapy with tumor-specific targeted therapies, e.g., therapies targeting Her2, c-Met or VEGFR [35–37]. These approaches are likely to be further developed and expanded in an effort to improve the still dismal perspectives of patients suffering from metastatic esophagogastric cancer.

In conclusion this phase 2 trial has demonstrated that folic acid and vitamin B12 supplementation does not improve the RR, PFS or OS of cisplatin and gemcitabine in patients with AEGC. We here show that the combination of gemcitabine/cisplatin is a reasonable alternative treatment schedule for patients with AEGC in case of dysphagia or preexistent neuropathy.
